# University student perspectives on antimicrobial peptide use in farm animals

**DOI:** 10.1371/journal.pone.0309986

**Published:** 2024-12-05

**Authors:** Amalia Urloiu, Barsha Shah, Jillian Hendricks, Katherine E. Koralesky, Daniel M. Weary, Adam Shriver

**Affiliations:** 1 Animal Welfare Program, Faculty of Land and Food Systems, The University of British Columbia, Vancouver, British Columbia, Canada; 2 Augustana University, Sioux Falls, South Dakota, United States of America; 3 Bristol Veterinary School, Langford House, Langford, United Kingdom; 4 W. Maurice Young Centre for Applied Ethics, School of Population and Public Health, The University of British Columbia, Vancouver, British Columbia, Canada; 5 The Harkin Institute for Public Policy and Citizen Engagement, Drake University, Des Moines, Iowa, United States of America; University of Birmingham, UNITED KINGDOM OF GREAT BRITAIN AND NORTHERN IRELAND

## Abstract

Growing awareness of antimicrobial resistance (AMR) in livestock production has led to calls for the development of alternatives such as antimicrobial peptides (AMPs) that are also able to combat infectious diseases in farm animals. A critical step in the development of AMPs is to understand people’s perspectives towards this technology to avoid misalignment with societal expectations. The aim of this study was to investigate university student perspectives of AMP applications in farm animals as alternatives to antibiotic use. We interviewed 20 university students and, using thematic analysis, identified six themes: 1) Initial knowledge, including knowledge of antibiotics and initial impressions of AMPs; 2) Human wellbeing, including the effects of food and animal health on public health, the importance of addressing AMR, and cost for farmers; 3) Animal welfare, including animal health, welfare, and production, and continuation of contentious farm practices; 4) Perceived naturalness of AMPs, including biocompatibility and comparing same and different species transfer of biological material; 5) Unforeseen consequences of AMPs, and the importance of researching unintended consequences of novel technologies; and 6) Public acceptance of AMPs, including trust and lack of awareness. In summary, participants viewed AMPs positively as an alternative to antibiotic use in farm animals to address AMR. However, key concerns centered around unintended harmful effects for food systems, public health, and animal welfare, which may impact public acceptance of AMPs in animal agriculture.

## Introduction

Antibiotics can help treat infectious diseases in farm animals, and at a subtherapeutic dose (defined as a dose too low to treat established infections; sometimes added to animal feed) can benefit growth and feed efficiency [1]. For all these reasons, antibiotics are commonly used on farms. For example, in 2016, 87.5% of feedlots and 95.5% of swine farms in the United States (US) administered antibiotics through feed, water, or injection [2, 3]. Some authors predict that antibiotic use will continue to increase, projecting a 67% increase in global use in food animals from 2010 to 2030 [4].

Despite the benefits of antibiotics, widespread use has given rise to antimicrobial resistance (AMR), a growing public health issue threatening the efficacy of treatments for diseases and infections caused by microorganisms such as bacteria that have evolved to persist against antimicrobial medications [5]. In 2019, the US Centers for Disease Control and Prevention reported over 2.8 million antibiotic-resistant infections in humans, resulting in over 35,000 deaths [6]. Administration of subtherapeutic doses of antibiotics in animal feed may be especially problematic in promoting the selection of resistant bacteria and causing changes in gut microbiome associated with an increase in the abundance and diversity of AMR genes [7]. Furthermore, animals do not metabolize all antibiotics that are consumed; the residuals are excreted, leading to the presence of AMR in the environment [8]. For example, [9] suggested that subtherapeutic administration of antibiotics can promote AMR in soil bacteria if improper manure dispersal occurs. Resistant bacteria can enter human exposure pathways through contamination of soil, water and air [10], reducing the efficacy of medically important drugs and limiting treatment options for human patients [11]. Exposure to farm animals has been linked to the presence of AMR in humans; for example, a study in Iowa found that humans with swine exposure exhibited a higher risk of hosting *Staphylococcus* bacteria resistant to antibiotics [12].

Public concern regarding antibiotic use is high; one study reported that more than 90% of US participants felt that antibiotic use on dairy farms posed a threat to human health, and 71% indicated that they would pay more for milk produced from cows that had not been treated with antibiotics [13]. In another study, German, Italian, and US participants expressed criticism towards antibiotic use on livestock farms [14]. In the US, participants were in favor of dairy farmers treating sick animals, when necessary, but worried about the consequences of antibiotic treatment on human and environmental health and instead preferred preventative strategies [15]. Furthermore, people in the United Kingdom believed that pork labelled as “raised without antibiotics” was produced following higher animal welfare standards [16]. This belief is a misconception; animals reared without antibiotics had an increased risk and severity of disease due to bacterial infections [17]. Overall, these findings indicate that public perceptions of farm animal welfare may be improved if alternatives to antibiotic treatment are implemented.

Antimicrobial resistance and public concern regarding antibiotic use in agriculture suggest the need for alternative methods of treating infectious disease in farm animals [18]. Research on antimicrobial stewardship in livestock farming has focused on interventions that reduce the use of antibiotics [19]. While most dairy farmers stated that they were trying to reduce antibiotic use [20], farmers also believed the health of their animals may be compromised if they did not have access to treatment methods [21, 22]. Therefore, alternatives to antibiotic treatment are needed.

Antimicrobial peptides (AMPs) may serve as one such alternative. AMPs are small chains of amino acid residues that form part of an organism’s natural immune system and can target a range of microorganisms, including antibiotic-resistant bacteria [23, 24]. AMPs are able to destroy bacterial cells by forming ion channels or transmembrane pores and thus can penetrate the cell membrane [25]. As such, AMPs may address a variety of issues in agriculture, including disease resistance in plants, AMR and antibiotic residue in aquaculture, and over-use of antibiotics that are common in livestock production, including those associated with bovine mastitis [26].

As a novel technology, AMPs may be viewed as contentious. History has shown that public acceptance of other novel forms of biotechnology applied on farms (such as genetic modification) can be low [27]. A review concluded that “bioactive” technologies raise concern among consumers about unpredicted effects and uncontrolled use in agriculture [28]. In a focus group study, public perceptions of technologies that allowed the study of genomes (e.g., proteomics and functional genomics) were more positive than the use of genetic modification and gene drives in animal production systems [29]. Studies investigating public views about plant biotechnology [30] and gene drives for pest control [31] revealed environmental and ethical concerns, and questions about scientific uncertainty and technology development. These results suggest that members of the public express an array of questions and concerns when faced with novel biotechnology; understanding public views may help inform the development and trajectory of new methods [32].

To our knowledge, there is no existing literature on public perspectives of AMPs use in agriculture. Due to the complexity and novelty of AMPs, describing this technology to the public may be difficult. Some university students have a scientific background that should help in understanding the technology. As a first step in understanding broader public perspectives towards AMP use in farm animals, we aimed to describe the perspectives of university students, including their initial impressions and questions about the technology.

## Materials and methods

### Ethics approval

This study was approved by The University of British Columbia’s Behavioural Research and Ethics Board (Protocol no. H22-01872-A001) with approval of the Augustana University’s Institutional Research Ethics Board.

### Study participation

We recruited a convenience sample of 20 participants for this study, 10 from Augustana University (United States) and 10 from the University of British Columbia (Canada), between September 14, 2022, and April 30, 2023. These universities were chosen due to the authors’ affiliation and familiarity with the university campuses and departments, which facilitated participant recruitment. The study was advertised through physical posters distributed on both campuses and via emails directed to students from professors who were contacted via a staff directory to request advertising the study to their students. Department chairs of religion, psychology and biology were contacted at Augustana University. Participant recruitment at the University of British Columbia (UBC) also utilized a Facebook advertisement posted through the university-sanctioned account of the UBC Animal Welfare Program (available at https://doi.org/10.5683/SP3/KFFOIZ). To join the study, prospective participants were instructed to contact the researchers by the email provided in the study advertisement. Prospective participants were required to provide their official university student email as evidence of their enrollment at Augustana University or UBC. The sample selection criteria were not constrained to any year-level or department. A $10 Starbucks gift card was provided as compensation for a completed interview to incentivize participation. All participants provided their signed consent in a consent and study information document provided by email (available at https://doi.org/10.5683/SP3/KFFOIZ).

### Data collection

Interview duration ranged from 20 to 30 minutes. BS conducted Augustana interviews (n = 10) and AU conducted UBC interviews (n = 10). At the initial stage of the interview, participants were asked about their educational background, including program and year of study. Interview questions followed a semi-structured interview guide, probing participant knowledge and views regarding antibiotics and AMPs (Table 1; full interview script and guide available at https://doi.org/10.5683/SP3/KFFOIZ). The interview guide was developed using open-ended questions that the researchers felt would facilitate high quality responses (i.e., beyond yes/no answers) but attempted to avoid influencing participants’ answers (e.g., avoiding questions that framed technologies as ‘good’ or ‘bad’). Interviews are ideal for rich data collection and more detailed analysis of themes which explore the contextual dimensions that may influence a phenomenon of interest [35]. We reached data saturation, whereby we observed redundancy in the topics and ideas discussed throughout consecutive interviews [33, 34]. In qualitative research, sampling procedures are typically emergent rather than predetermined, subject to change based on information yielded from co-occurring data collection and analysis [33, 35].

**Table 1:** Summary of the questions (Q) in the semi-structured interview guide.

Questions	
Q1.	What is your educational background—what are you studying and what is your level of education?
Q2.	What do you know about antibiotics?
Q3.	How do you feel about the use of antibiotics in farm animals?
Q4.	Have you heard of AMPs? (If answers yes) Could you describe what you know about this? (If answers no) What do you think AMPs are?*
Definition:	There is a potential alternative to antibiotics called AMPs. AMPs are part of an organism’s immune system, and the AMPs can be transferred from one species to another to help the target species become resistant to a disease. AMPs are less likely to lead to resistance.
Q5.	What are your thoughts about AMPs?
Q6.	How do you feel about using AMPs in farm animals as an alternative to antibiotics? Configure your response in SWOT analysis format, describing potential Strengths, Weaknesses, Opportunities and Threats.**
Q7.	What questions do you have about AMPs?
Q8.	Where do you see AMPs in the next 10 years?

*Note: An AMP definition was given after Q4, regardless of how the participant responded.

**Note: Participants were given time to write out responses for the Q6 SWOT analysis.

### Data analysis

The transcripts were developed and anonymized using the transcription feature built into Zoom (Zoom Video Communications Inc, San Jose, CA, USA) and NVivo (version 12; Lumivero, Denver, CO, USA). We analyzed the transcripts using thematic analysis, a process that identifies, interprets and presents patterns, themes or concepts within the data [36]. First, authors BS and AU were responsible for initial coding of the interview transcripts. AU coded the transcripts from UBC interviews, and BS coded the transcripts from Augustana interviews. Initial coding involves a thorough examination of data to discover keywords, patterns, themes, or concepts before formal analysis [36]. Initial codes were developed by reading through the data and assigning descriptors in the form of short summarizing phrases or words to pieces of text in the transcripts. The initial codes were collated and organized collaboratively to develop a thematic codebook [36]. This process involved ongoing refinement, with the codebook guiding data coding and, in turn, influencing the codebook’s evolution as new themes and details emerged from the data. The preliminary codebook contained titled themes, well-defined codes, and subcodes, all established before assessing inter-coder reliability.

To evaluate inter-coder reliability, JH coded one Augustana and one UBC transcript independently. JH then met with AU and BS to review and address differences in the assigned codes and to develop subsequent versions of the codebook, focusing on improving codes that appeared redundant or had ambiguous descriptions, causing inconsistencies in coding. After researchers had reached agreement, BS and AU re-coded all transcripts using the finalized codebook (available at https://doi.org/10.5683/SP3/KFFOIZ). This reflexive approach of inter-coder reliability aligned with our iterative coding method and acted as a crucial measure of reliability to ensure consistent interpretation of the codebook and resultant replicability of the coding process [34, 37].

**Fig 1:**
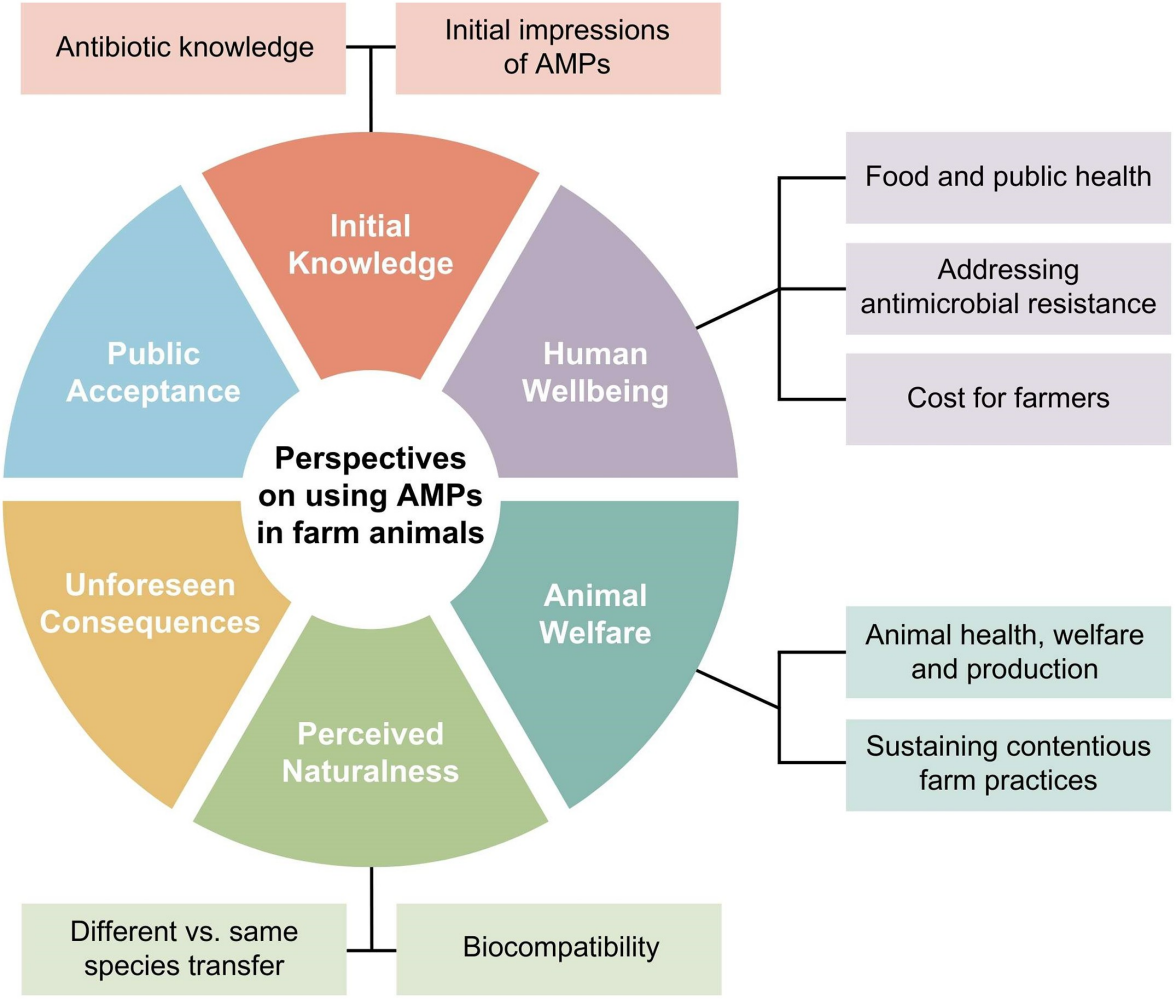
Thematic map developed from the university student interviews (n = 20) investigating participants’ perspectives around the use of AMPs in farm animals as an alternative to antibiotics. Pie-slice shaped colored sections with bold white text illustrate the six themes developed using inductive thematic analysis. Colored boxes with black text show subthemes.

Themes were developed from relevant codes and mapped to illustrate relationships (Fig 1). Illustrative quotes from the interview transcripts are shown below with the identifiers assigned to participants; quotes from participants from Augustana began with “AU” and those from UBC began with “UBC”, in each case followed by the arbitrary participant number (P1 to P10). Brackets are used to indicate grammatical corrections to improve clarity.

## Results and discussion

A high proportion of participants from both universities were in biology-related majors. The next most common major was philosophy at UBC and psychology at Augustana (see https://doi.org/10.5683/SP3/KFFOIZ).

We identified six themes (Fig 1): 1) Initial knowledge, including knowledge of antibiotics and initial impressions of AMPs; 2) Human wellbeing, including the effects of food and animal health on public health, the importance of addressing antimicrobial resistance (AMR), and cost of AMPs for farmers; 3) Animal welfare, including the intrinsic value of animal welfare, animal health and production and sustaining contentious farm practices; 4) Perceived naturalness of AMPs, including biocompatibility and comparing same and different species transfer of biological material; 5) Unforeseen consequences, including the importance of researching unintended consequences of novel technologies; and 6) Public acceptance of AMPs, including trust and lack of awareness.

### Initial knowledge

#### Antibiotic knowledge

Only two participants from Augustana University, and none from UBC, reported having prior knowledge about AMPs, but all participants said they had knowledge of antibiotics, and many brought up the issue of AMR before this was mentioned by the interviewer. Participants drew upon examples of usage in farm animals as well as humans when prompted to describe antibiotics.

#### Initial impressions of AMPs

When asked to develop their own conjectures of what AMPs could mean, participants suggested that the “antimicrobial” term referenced combatting pathogens. For example, AUP2 stated, “I would imagine it’s some sort of chemical to ward off harmful microbes”. UBCP4 expressed a similar idea: “Antimicrobial to me is like you would prevent microbes, and like parasites and diseases in stuff.”

A few participants also related the “antimicrobial” term to antibiotics and guessed that AMPs may be an umbrella term or offer a similar function as antibiotics, e.g., UBCP1: “It sounds similar to an antibiotic. I mean, maybe like a more general term name, like microbial, microbiology? […] maybe like an umbrella term for something that includes antibiotics”. Some participants with knowledge of peptides described AMPs as proteins that work to combat microbes, e.g., AUP3: “so peptides are what proteins are made from, so I guess like proteins or like pieces of a protein that fight microbes”. Thus, while our participants generally did not have pre-existing knowledge of AMPs, they associated it with antibiotics due to a similarity in nomenclature, and used their understanding of basic biology to draw inferences on what AMPs could be.

### Human wellbeing

#### Food and public health

Participants discussed AMPs entering the food system through livestock and possible influences this could have for public health. Participant UBCP6 stated that “[…] food safety is number one for the public”, and felt that many people would be concerned about AMPs entering the food system even though they did not personally view this as their concern:

UBCP6: “I know there’s a lot of discussion about having drugs in your body because it’s in the food chain. […but] as long as it’s not an active ingredient in what I’m eating, it really doesn’t matter to me.”

Participants compared the effects of antibiotics versus AMPs on food and public health. Participant AUP10 stated “you can put it [AMPs] potentially […] in the food population, and it could cause way less harm than putting antibiotics in. Or it could potentially have some upsides, like human use.” Similarly, AUP4 stated “given that bacteria will evolve pretty quickly, I can see where [AMPs] would be really useful for breeding for food stock” expressing positive feelings over use of AMPs in animals destined for human consumption. AUP1 emphasized the importance of the health of food animals, stating “we’ve got something other than antibiotics that we can use to prevent animals that we’re going to eat from getting sick [and that] is definitely nice.”

Ambivalence towards potential benefits and threats of novel technology was documented in a review by [38]. Potential benefits to human health, for example, reducing diseases in non-human animals to prevent transmission of diseases to humans, was the primary reason that participants supported genome editing in non-human animals [38]. However, some participants also discussed potential unknown effects of modified organisms on human health [38]. Previous research on public attitudes towards novel biotechnologies used in livestock have identified similar concerns. A study on consumer attitudes, ethical considerations and perceptions of human health implications towards gene editing of livestock found that consumers require either discounts in price or added benefits to consider consuming GE animal products [39]. In another study on public perception towards genetic modification in animal production [29], participants expressed reservations about genomic technologies seen as "artificial," particularly genetic modification (GM) and gene drives. They were concerned that these technologies could lead to unintended effects with serious implications for human health [29].

While discussing opportunities and threats of AMPs, one participant described the interconnected wellbeing of humans, non-human animals and the environment:

UBCP8: “The downstream effect of improved health is diffused at all aspects of the biosphere. So not just animals, but humans, and therefore the nature that they rely on. If there’s not as much illness (due to AMPs), ideally, the entire system has a ripple effect of getting healthier. At the same time, […] if a person or an animal is being misused, then the whole system is likely suffering.”

This conceptualization relates to One Health, described as a multidisciplinary approach for improving public health by accounting for the interconnected relationship of human health, animal health and ecosystem health [40]. One Health approaches are increasingly applied to work on public and livestock health [41–43], as discussed in the following sections.

#### Addressing antimicrobial resistance

Participants supported the implementation of AMPs to address AMR in livestock agriculture to avoid subsequent harm to public health. One participant stated, “I think one of the strengths [of AMPs] is simply that we aren’t using antibiotics anymore on animals, and we want to prevent antibiotic-resistant bacteria from developing, that is a definite strength in and of itself” (AUP1). However, a few respondents expressed that efficacy of AMPs compared to antibiotics must be achieved alongside reduced risk of resistance, e.g., “[…] if there’s less of a chance for the bacteria to become resistant to them, it looks like the best possible option as long as they’re just as effective as an antibiotic […]” (AUP10). Findings from previous work have supported that AMPs could effectively regulate the host’s immune response to infections and therefore replace at least some antibiotic use [44, 45]. The impact of AMR on human health appeared to heighten the importance of AMPs among our participants. For example, UBCP6 described their support of anything that would establish immunity in an animal without increasing the risk of antibiotic resistance in humans. They went on to say, “I think that anything that avoids those risks is ideal, and I would agree with it preemptively”. Thus, participants recognized that AMR in livestock can put human health at risk, and supported AMPs to reduce this risk.

Prior literature has demonstrated public concern around antibiotic usage in farm animals, public interest in alternative treatment options, and public belief that pervasive antibiotic usage poses a threat to public health [13–16]. Some work has investigated public perceptions of AMR. For example, one review reported that the public has incomplete knowledge about AMR in general and believes that their individual risk of AMR is low [46]. Awareness of AMR in animal agriculture varied across five European countries (Austria, the UK, Poland, Spain and Denmark), with Spanish participants being the least aware and Austrian participants the most [47]. Greater awareness and concern were associated with interest in alternative treatments to antibiotics, such as vaccines and AMPs [47]. Future studies investigating public acceptance of AMP use in farm animals should consider awareness of and concern for AMR.

#### Cost for farmers

Potential economic and production-related consequences of AMP usage in livestock were weighed in the interviews. In UBC transcripts, the impact of AMPs on production efficiency was linked to cost for farmers, particularly when related to disease spread if AMPs failed to be effective: “it could really hurt the livestock […] if an infection went viral […] it can really have drastic financial consequences for the farmer” (UBCP7). When considering the benefit of being able to use AMPs more frequently than antibiotics, without the same risk of AMR, another participant stated, “[…] they [farmers] could use it again, and help the animal to produce again, […] and depending on the cost of stuff, that could be a strength” (UBCP2). These views are similar to those expressed by New York State dairy farmers who considered antibiotics to be a financial strain due to cost of medications and disposing milk from cows treated with antibiotics [22].

Participants considered cost as an important factor in the implementation of AMPs, including “getting over that bump of getting farmers to make the switch” (UBCP5) to AMPs. One participant believed that “they [AMPs] will take off if it’s effective and doesn’t drive up the cost” (UBCP10). However, participants were also concerned about constraints on how AMPs would be generated or marketed, and how this might affect access for farmers:

UBCP3: “With patents on the corporatization, and big Pharma and capitalism, we don’t know if it’ll be accessible even to farmers. Maybe farmers will become more reliant on it, and then it’ll skyrocket in price, and then we won’t be able to use it anymore.”

AUP10 asked about the accessibility of AMPs compared to antibiotics, “Production-wise compared to antibiotics, which of the two are easier to produce, have less costs?”, indicating that these factors might affect how well AMPs are utilized. Concern about the cost of AMPs needs to be seen in the context of previous work showing willingness to pay for food products that have positive animal health and welfare attributes; for example, a study on US citizens described a willingness to pay more for cheddar cheese that came from farms that did not use antibiotics, required pasture access, and used pain relief during dehorning [48]. Given our participants’ concern for the cost of AMPs, accessibility of this novel technology to both farmers and consumers should be a priority for those developing and implementing it.

### Animal welfare

#### Animal health, welfare, and production

Participants, particularly those from UBC, described concerns about animal welfare as critical for their support of AMP use in animals, including concerns relating to pain or suffering experienced by the animals. One participant shared that they believed “if [AMPs] work to relieve [animals] of whatever pain they’re experiencing in the same way, then I think it’s good” (UBCP2). Another participant explained, "I think [AMPs] could be a great tool, because obviously we don’t want farm animals to be suffering from disease […] I think AMPs, if it’s effective, and if safe, could be more ethical or better for their welfare […]” (UBCP3). When discussing the reasons for accepting or rejecting the implementation of AMPs in farm animals, several UBC participants expressed that animal welfare was not just a personal concern, but a concern that would be reflected in the general public, e.g., “I think that the public’s understanding of animal welfare, and its importance in their eyes, is definitely growing” (UBCP6).

Animal welfare is becoming important to the public and can affect their views of livestock production systems [49]. Some studies investigating public attitudes towards gene editing or genetic modification in livestock, such as polled cattle, documented concerns about pain and the welfare of animals, and demonstrated willingness to pay more for products utilizing polled genes to improve welfare [39, 48, 50]. A study by [29] reported that most participants considered animal health as integral to animal welfare, and that genomic technologies were seen as valuable if they could enhance both health and welfare.

Discussions around production were connected to farm animal welfare by UBC participants. Some participants considered farm animal welfare and production to be combined goals of implementing a biotechnology like AMPs, e.g., “If there’s research saying it’s better, then I don’t see why it [AMPs] wouldn’t be heavily implemented. I do think ultimately people want their farm animals to be healthy and well and producing” (UBCP2). Other participants considered animal welfare as integral to achieving successful production, e.g., “I think that especially with livestock, while the production levels are important for farmers, I think that in order to have good production levels, you need healthy livestock, so I think that their first priority should be the animal welfare” (UBCP4).

The examples above reflect the view that animal welfare and production are positively correlated. However, other responses showed that some were concerned that AMPs might also be used for production benefits even at the expense of animal welfare. These participants supported the use of AMPs only for improving the health or wellbeing of sick animals but were against the implementation of AMPS for growth and production. One participant stated, “I think when [AMPs] go outside of welfare, and more for increasing production rates, and the size of the animal, and doing it to get more money, I’m against that” (UBCP4). Another participant stated this objection more strongly: “Basically, humans get more out of their farm animals. […] But it doesn’t sound very ethical. The fact of the matter is that this market is humans using animals for their benefit.” (AUP9). In a qualitative study on US public views on the use of antibiotics in livestock, [15] found that some participants were only supportive of therapeutic use of antibiotics and were against usage for prophylaxis or growth promotion. In another study, public perceptions of gene editing in cattle were more negative when the technology was described as increasing muscle tissue growth (an application resulting in more meat) versus when described as reducing heat stress or producing hornless animals (an application that improves animal welfare) [51]. In our study, some participants felt that animal welfare and production could be improved simultaneously by AMPs, but others rejected the idea of implementing this technology solely to improve production, especially if this increased productivity was associated with reduced welfare.

#### Sustaining contentious farm practices

Participants expressed concerns around AMPs sustaining contentious farm practices. Although improving herd health was generally seen as a strength of AMPs, some participants identified that this technology could be used to maintain overcrowded housing. For example, UBCP1 stated that “[AMPs] could be used to justify still having animals in close proximity, maybe like at worst, could have harms to animal welfare, because there’s no longer those concerns about animals being sick.” In addition, AUP8 argued that “This drug could help animals live longer, and if they’re in bad conditions, it would be unethical to extend their life. And then maybe people would take advantage of that and have too many animals in one area where it’s overcrowded because they’re all reproducing more.” These quotes reflect the concern that the technology may allow farms to avoid solving the root causes of overcrowding issues in livestock production.

Other participants expressed a general objection to the use of technology to sustain poor production practices: “If [AMPs] just continue to support the status quo I’m likely not going to be in favor, and I hope there’s a growing amount of people who are also not going to be in favor” (UBCP8). A few participants viewed AMPs as a band-aid solution for general herd health issues and thus a distraction from solving contentious farm practices at the core of these issues, e.g., “[…] the risk of this being used as kind of a mechanism to continue unsafe farming practices; we’ll just deal with the problem when it comes. But I think in the perfect world that we would want to avoid putting animals at risk before having to fix the issue afterwards” (UBCP9). A study in the Netherlands by [52] reported that participants expressed similar concerns around allowing systemic farm issues to prevail as a result of gene editing in livestock, and a survey of North Americans found that some viewed gene editing as a band-aid for larger issues in farming [53]. Others have expressed the concern that novel biotechnologies will be used to alter livestock to fit better within unsuitable agricultural systems [38, 54].

### Perceived naturalness

#### Biocompatibility

Many participants perceived AMPs as more natural than antibiotics after listening to the AMP definition provided (Table 1), often attributing this to the description of AMPs being part of an organism’s immune system. This perceived naturalness resulted in participants believing that AMPs could be more biocompatible (i.e., integrating into the host’s immune system with low risk of harm to the host) compared to antibiotics and result in fewer side-effects, e.g., “[…] considering [AMPs are] something that’s already present in organisms naturally, I think that could help ensure that they don’t do any harm to the organisms if they’re used” (UBCP3). This perception often led to greater acceptance of AMP implementation, e.g., “I keep saying natural, but it just does seem more in line with the body’s natural processes. I think we’re just starting to realize that those things have a lot of value, so I could see [AMP] use becoming prevalent” (UBCP1). AMPs have displayed low cytotoxicity, promising cell selectivity, low resistance and a wide spectrum of antimicrobial biological effects in mammalian cells that have led to their consideration as an alternative to antibiotics in livestock agriculture [23, 26, 44]. One participant described AMPs as more natural than antibiotics and that it was a better alternative; AUP7: “I would assume it’s a better alternative since they’re made naturally, and antibiotics are formed in the lab […].” This perspective around naturalness and biocompatibility demonstrated the ability of participants to envision considerations for AMP usage beyond the limited information provided in the interviews. The concept of naturalness and its role on attitudes towards novel biotechnologies has emerged in previous studies; the concept appears to be deeply rooted in value and belief systems and thus should be considered by developers of these technologies [55].

### Different vs. Same species transfer

Some participants who discussed the naturalness of AMPs expressed reservations around transferring AMPs between organisms of different species. Transfer between different species appeared to introduce trepidation around potential unknown effects, resulting in a preference for transfer within species, e.g., “Instead of passing it from one species to another, because I feel there’s a huge variety of unknown in that, maybe you could pass it from the same species” (UBCP4). Previous studies on public attitudes towards genetically modified foods have identified a preference for cisgenic compared to transgenic foods, due to greater perceived naturalness [56, 57]. One participant (UBCP10) in the current study shared that transfer between different species could reduce public acceptance of AMPs: “Some people may be afraid of it—sounds almost like a GMO a little bit, just because it’s trading between species.” Thus, while some of our participants favored AMPs for their greater biocompatibility compared to antibiotics, others expressed skepticism about transferring material between different species.

### Unforeseen consequences

Many participants discussed unforeseen consequences as a concern around the use of AMPs in farm animals and related this to food system and human health. Participants expressed that these concerns may be alleviated with more research, e.g., “Personally, I would be okay with them being used if there was research that was being done to show that they are effective and that they are safe. But I think I would kind of need that reassurance” (UBCP3). This reassurance was connected to safety as well as monitoring long-term outcomes, as explained by participant UBCP4: “I do think it’s very important, and I think that new antibiotics and new AMPs are a trial-and-error thing, and I think that in order to know if it’s successful or not, you need to look at it from a long-term perspective, because you need to be able to understand how the body reacts to it over a long period of time, not just the short fix of getting rid of the infection.”

In a study on public attitudes towards genetically modified polled cattle [50], participants also expressed uncertainty around implementing the novel biotechnology due to the possible long-term unintended consequences for the cattle. A review investigating consumer acceptance of novel agricultural food technologies similarly observed that participants expressed concern around long-term and unforeseen consequences of technology for the environment or public health [28]. Thus, fear of unintended long-term effects of novel biotechnologies, for both livestock welfare and public safety, appears to be a prevailing theme across this study and the prior literature [13, 27–30, 39, 47, 50].

### Public acceptance

Most participants were unfamiliar with AMPs before this study. UBC participants associated their unfamiliarity to lack of awareness among the public, e.g., “I feel like for the general public, maybe just based on how it’s kind of confusing me, but [AMPs] may not be so widely understood, and it might not be so easy to implement it in every farming practice” (UBCP9). These participants associated their lack of awareness with lack of public acceptance for AMP implementation, describing that a barrier to “getting [AMPs] applied would be a general lack of public knowledge and likely resistance to change” (UBCP5). UBCP5 elaborated how fear of the unknown may inhibit public acceptance of AMPs as a novel technology, explaining that “even though farm animals currently get antibiotics, and the plants get covered in pesticides, those aren’t new things so they’re kind of just accepted; but I think introducing a new thing would bring up a lot of fear in people, especially if they don’t understand the science behind it.”

Some participants inquired how much is known about AMPs and related that to lack of acceptance: “If there’s something that goes wrong, how is the public going to react? They’re going to say; I don’t want AMPs being in my food” (AUP1). Overall, participants of this study believed that the novelty of AMPs, and lack of public awareness and scientific understanding of how this biotechnology functions, could deter efforts to implement AMPs in animal agriculture. Compared to experts (i.e., biologists), members of the public in a prior study perceived medical and agricultural biotechnology to be more risky, more harmful and less useful, and were concerned with how much science knew about the technology [58]. A review by [59] similarly suggested that a higher level of education, awareness and knowledge of genetically modified food was positively correlated with public acceptance of the biotechnology. Open, transparent and accessible communication about AMPs, with a focus on concise and understandable explanations, as well as opportunities for reciprocal inquiry and feedback between the public and professionals, may promote public acceptance [27, 28, 30, 32].

### Emerging questions about AMPs

Table 2 summarizes questions raised by participants during the interview. Most often these questions emerged in response to Q7 of the interview guide: “What questions do you have about AMPs?”. Questions posed by participants were coded into seven sub-categories. Preparing responses to these questions or integrating the answers into a more in-depth description of AMPs may help future communication efforts regarding the use of AMPs in farm animals [60, 61]. Characterization of questions commonly posed by the public has been used as a communication tool to provide information about a variety of topics, including climate change [62] and COVID-19 [63]. The questions in our study can be similarly considered for other novel biotechnologies when developing materials that aim to bridge knowledge gaps and target areas of interest for the public.

**Table 2:** Questions (Q) posed by participants (n = 20) about AMPs coded into seven sub-categories.

Questions	
Q1a.	What is the method of application for AMPs?
Q1b.	How invasive is the procedure for the animal?
Q2.	What is the current stage of research and implementation for AMPs?
Q3.	How effective are AMPs in comparison to antibiotics?
Q4.	How do AMPs work biologically to prevent or treat disease?
Q5.	How are AMPs being applied, i.e. to what end or purpose?
Q6.	What is the potential for long-term or downstream side effects from AMPs?
Q7.	Are AMPs being implemented in humans?

### Study limitations and future directions

Our study is, to our knowledge, the first to investigate perspectives towards AMP use in agriculture. Participants expressed a high level of curiosity and concern regarding AMPs, as evidenced by the questions posed during the interviews. This underscores the importance of addressing public inquiries and integrating the answers into explanations to bridge knowledge gaps and communicate the benefits and risks of AMPs.

An important limitation is our use of a convenience sample of students from two universities; findings should be considered within this context. That said, this sample was not intended to be representative of a larger population. A limited sample size of the current study was selected to allow for an in-depth view of participant reflections [35]. The results of the current study could also be used to inform the development of a quantitative survey questions targeted a larger and more diverse sample.

A specific limitation of our sample of university students is that many had specific training in topics related to the issue, including participants majoring in biology and philosophy. The perceptions of ethical issues relating to novel technologies may well differ for participants with these educational backgrounds compared to those of the broader public. For example, a previous study reported that participants with a college or university degree held greater optimism towards nanotechnology in the food system [64]. Similarly, a case study of the Chinese public found education to be the most important factor in determining attitudes towards agricultural biotechnology, with more educated people holding more optimistic attitudes [65]. Thus, participants in our study likely held more optimistic views towards AMPs than people outside of a university setting. We encourage future research to investigate perspectives of the broader public, including individuals from diverse educational, cultural, and socioeconomic backgrounds.

An important result is that few of our participants had previously heard of AMPs; this serves as both a strength and a limitation of this study. On the one hand, we were able to address perspectives that had not yet been influenced by pre-existing attitudes, and thus capture how people respond when first exposed to the idea. On the other hand, our description of AMPs likely had a framing effect. The participant responses described above had a slight tendency to be positive, especially in seeing AMPs as an alternative to antibiotics with the potential to reduce AMR. Previous research has shown how framing technology affects responses, including frames related to benefits and risks [e.g., 66]. We framed AMPs as applicable in farm animals; previous research has found that experts and the public perceive biotechnology used in agriculture as more risky than medical applications [27]. Future research should compare public perspectives towards different AMP applications to understand the effects of different frames on these perspectives.

Given the challenge of low familiarity with AMPs, future investigations should consider giving participants more opportunities for learning and asking questions. This could include small focus group discussions or two-way dialogues between the public and AMP stakeholders. Longitudinal studies could be beneficial, allowing participants time to reflect on the technology and to better assess the impact of any interventions on public perceptions.

The study should also be viewed within the broader context of understanding how the public reacts to agricultural biotechnologies more generally. Previous research has identified several factors that may shape public perceptions, including perceived risks and benefits, knowledge and education, cultural values, and purchasing behavior [27, 29, 32, 58, 59]. Understanding these factors can inform the development of effective communication strategies that address public concerns and promote informed decision-making. Future research can further explore how public attitudes towards AMPs connect to these more general frameworks.

Finally, given the general lack of familiarity with AMPs, it is crucial that experts also explore the ethical implications of this technology. As the technology continues to be studied, additional research should be conducted on the potential impact of AMPs on animal welfare, environmental sustainability, and human health to allow for better informed decisions about future use of AMPs.

## Conclusions

This study explored student perspectives regarding AMP applications in farm animals. We found that most participants lacked specific knowledge of this technology, but most had a basic understanding of antibiotics and used this to draw inferences on the definition of AMPs. Concerns among participants encompassed potential impacts on human health through food systems and direct consumption of animal products, as well as considerations for animal welfare. Economic factors, such as production efficiency and disease prevention, were seen as potential advantages of AMPs, but concerns about cost and accessibility were also raised. The issue of AMR was recognized, with AMPs seen as a potential solution. Participants tended to perceive AMPs as more natural compared to conventional antibiotics. While participants were largely optimistic towards AMPs, many were pessimistic about the public’s ability to gain awareness and trust around AMPs as a novel biotechnology affecting the food system.
